# First-Row Transition Metal Doping in Calcium Phosphate Bioceramics: A Detailed Crystallographic Study

**DOI:** 10.3390/ma10010092

**Published:** 2017-01-23

**Authors:** Guillaume Renaudin, Sandrine Gomes, Jean-Marie Nedelec

**Affiliations:** Université Clermont Auvergne, CNRS, SIGMA Clermont, Institut de Chimie de Clermont-Ferrand, F-63000 Clermont–Ferrand, France; sandrinerenaudin@neuf.fr (S.G.); jean-marie.nedelec@sigma-clermont.fr (J.-M.N.)

**Keywords:** apatite, doping, cationic substitution, bioceramics

## Abstract

Doped calcium phosphate bioceramics are promising materials for bone repair surgery because of their chemical resemblance to the mineral constituent of bone. Among these materials, BCP samples composed of hydroxyapatite (Ca_10_(PO_4_)_6_(OH)_2_) and β-TCP (Ca_3_(PO_4_)_2_) present a mineral analogy with the nano-multi-substituted hydroxyapatite bio-mineral part of bones. At the same time, doping can be used to tune the biological properties of these ceramics. This paper presents a general overview of the doping mechanisms of BCP samples using cations from the first-row transition metals (from manganese to zinc), with respect to the applied sintering temperature. The results enable the preparation of doped synthetic BCP that can be used to tailor biological properties, in particular by tuning the release amounts upon interaction with biological fluids. Intermediate sintering temperatures stabilize the doping elements in the more soluble β-TCP phase, which favors quick and easy release upon integration in the biological environment, whereas higher sintering temperatures locate the doping elements in the weakly soluble HAp phase, enabling a slow and continuous supply of the bio-inspired properties. An interstitial doping mechanism in the HAp hexagonal channel is observed for the six investigated cations (Mn^2+^, Fe^3+^, Co^2+^, Ni^2+^, Cu^2+^ and Zn^2+^) with specific characteristics involving a shift away from the center of the hexagonal channel (Fe^3+^, Co^2+^), cationic oxidation (Mn^3+^, Co^3+^), and also cationic reduction (Cu^+^). The complete crystallochemical study highlights a complex HAp doping mechanism, mainly realized by an interstitial process combined with calcium substitution for the larger cations of the series leading to potentially calcium deficient HAp.

## 1. Introduction

Biphasic Calcium Phosphates (BCP) are a promising class of bioceramics for applications in bone repair/replacement surgery. Calcium orthophosphates are the most widely-used bioresorbable ceramics [1], and are also used in pharmaceutical applications as a carrier for drug or gene delivery systems [2]. Calcium orthophosphates are present in the bones, teeth, and tendons of mammals [3]; namely the Ca_10_(PO_4_)_6_(OH)_2_ hydroxyapatite phase composing the mineral part of bones (45–70 weight percent; wt %), dentine (45–70 wt %), and enamel (~95 wt %) [4]. The mineral component of bone—i.e., bioapatite—is more accurately a nano-multi-substituted carbonated hydroxyapatite, containing about 7 wt % carbonate, ~0.9 wt % sodium, ~0.7 wt % magnesium, ~0.1 wt % chloride, ~0.03 wt % fluoride, potassium, and strontium, as well as trace levels of silicon (250 ppm), zinc (25 ppm), chromium (0.25 ppm), cobalt (0.2 ppm), and manganese (0.1 ppm) [5]. BCP ceramics are mainly composed of hydroxyapatite (HAp) mixed with a smaller amount of beta tricalcium phosphate (Ca_3_(PO_4_)_2_, β-TCP) [6]. The magnesium-containing form of β-TCP (named whitlockite Ca_3−*x*_Mg*_x_*(PO_4_)_2_ [7]), is found in biological calcifications: dental calculi, dental caries, urinary and salivary stones, arthritic cartilages, and soft-tissue deposits [8,9,10]. BCP ceramics take advantage of the difference in solubility between β-TCP (the more soluble when implanted in the body) and HAp (less soluble, with long-term bioresorbable properties) [3]. Another interesting aspect of BCP is the possibility to prepare multi-substituted ceramics—both HAp and β-TCP structures are known to accept a large variety of atomic incorporations—in order to improve their biological and/or mechanical behavior.

Doping (introducing a chemical species in small proportions) is an interesting way to tune BCP behavior for at least two reasons: (1) control of the HAp/β-TCP ratio and (2) improvement and design of the bioceramic properties. Nevertheless, our previous results on BCP doping demonstrated the necessity of carefully analyzing the composition of the doped ceramic in order to correctly understand its behavior. The mineral composition of BCP and the actual location of the doping element depend both on the doping element used and on the applied sintering temperature. The case of Sr substitution in BCP has highlighted the impact of the sintering temperature on the HAp/β-TCP ratio [11,12], and Mg substitution in BCP has underlined the effect of the sintering temperature on doping element location (either preferentially in HAp, or in β-TCP) [13]. The HAp crystal structure is usually described as accepting various and large amounts of substitutions; however the competition with β-TCP substitution has to be considered in order to perfectly describe the doped BCP ceramic. For example, a high sintering temperature for Sr-doped BCP stabilizes the Sr:HAp phase, whereas a high sintering temperature for Mg-doped BCP stabilizes the Mg:β-TCP phase (whitlockite). Such differences impact the biological behavior of the bioceramic, since the doping element can be located in the mineral phase with either low solubility (HAp) or high solubility (β-TCP). These two alkaline earth cases remain relatively simple to characterize, because only a calcium substitution mechanism has to be considered.

Our recent detailed study on Zn insertion in BCP has shown a more complex mechanism, with the incorporation of Zn^2+^ at the interstitial crystallographic site of the HAp structure, and the incorporation of Zn^2+^ by substituting calcium in the β-TCP crystal structure [14,15,16]. The HAp insertion mechanism is favored by a sintering temperature above 900 °C, whereas the β-TCP substitution mechanism is favored by an intermediate sintering temperature of around 700 °C, and lower sintering temperatures favor the location of Zn^2+^ on the amorphous shell of nano-sized undoped HAp crystals. This dependence on synthesis conditions explains some discrepancies reported in the literature, and implies different release kinetics in biological fluids. It is thus very important to fully characterize the prepared doped samples in order to predict and/or correctly understand their biological performance upon implantation. It is obvious that doping elements can be used to provide interesting new properties, but in the case of medical applications too large or too fast a release of the doping element in the body can also have dramatic effects. The release kinetics of Zn^2+^ in simulated body fluid has been shown to depend on the synthesis conditions [11]. Biological apatite contains several trace elements conveying biological properties [5]. The bio-inspired doping of synthetic hydroxyapatite can thus be used to tailor its biological properties, in particular by tuning the release amounts upon interaction in the body. Zinc plays an important role in the normal growth and development of the skeletal system; it inhibits osteoclast differentiation and promotes osteoblast activity [17,18]. Pure HAp presents a lack of antibacterial activity, which limits its use as a biomaterial. The incorporation of inorganic antibacterial cations is a prerequisite for medical applications. In the last decade, silver (Ag^+^), copper (Cu^2+^), zinc (Zn^2+^), and titanium (Ti^4+^) have been investigated to prevent microbial infections while preserving non-toxicity toward human cells at low concentrations [19,20,21,22,23,24,25,26]. Manganese influences the regulation of bone remodeling, and a Mn-doped hydroxyapatite coating has shown a beneficial effect on bone cells, with a greater osteocalcin production, in a similar way to that of the well-known Sr^2+^ doping effect [27]. Manganese doping involves smaller and less perfect HAp crystals (nanocrystal features resembling bioapatite) that should be more resorbable, and therefore more biocompatible [28]. Iron-substituted HAp magnetic nanoparticles can find applications as heating mediators in hyperthermia therapies [29,30], with a reported maximum doping level of 2.23 wt % Fe [31]. Other biomagnetic doped HAp nanoparticles, incorporating magnetic iron, cobalt, or nickel cations, can be considered to develop biological applications [30], and a recent study has shown the high blood compatibility of Mn^2+^ and Fe^3+^-doped nano-sized hydroxyapatite [32,33]. 

The recent review of Supova [1] presents the numerous papers published on the production of doped synthetic hydroxyapatite without reporting the temperature-dependent insertion mechanism highlighted for Zn^2+^ [15], and recently for Fe^3+^ [34]. In the present paper we report and compare the first-row 3d transition metal (from manganese to zinc) doping of BCP ceramics. Ionic incorporation into BCP will result in structural modifications of both HAp and β-TCP phases: lattice parameters variations due to the ionic radius and the location of the doping 3d metallic cations are determined from small variations in the relative diffraction peak intensities. Table 1 presents the ionic radii of the investigated cations (taken from [35]). The studied 3d metallic cations are clearly smaller than calcium cations, with a difference of 54% for the smallest Co^3+^ LS and 83% for Mn^2+^ HS when considering the common octahedral coordination. The results presented here are mainly obtained from Rietveld analyses of the X-ray powder patterns of the synthesized samples. Conclusions are based on previous detailed characterizations of the incorporation of zinc [14,15,16], iron [34], and copper (paper in preparation) in BCP, for which Rietveld analyses of both X-ray and neutron diffraction patterns were combined with X-ray absorption, Mossbauer and X-ray Photoelectron spectroscopies. The Zn-doping case has been deeply investigated: for intermediate sintering temperatures, Zn^2+^ cations are mainly incorporated into the β-TCP structure, substituting Ca^2+^ cations at the Ca4 and Ca5 crystallographic sites (corresponding to the “low density” column of the crystal structure [36]) and forming the Ca_3−*y*_Zn*_y_*(PO_4_)_2_ substitution solid solution with a decrease in the lattice parameters of the hexagonal unit cell in correlation with the smallest ionic radius of Zn^2+^. When using a higher sintering temperature, the Zn-doped β-TCP phase is destabilized to the benefit of a Zn-doped HAp compound. It has been undeniably demonstrated (using a set of experimental observations) that Zn^2+^ incorporates the HAp structure at an interstitial crystallographic site leading to the chemical composition Ca_10_Zn*_x_*(PO_4_)_6_(OH)_2−2*x*_O_2*x*_, with Zn^2+^ cations located along the hexagonal channel between two hydroxyl crystallographic sites, forming linear O-Zn-O entities. Subsequent studies concerning copper and iron have shown significant similarities (namely the temperature dependence of the doping element location and the interstitial mechanism associated with the HAp structure) and have also shown interesting specific behaviors. In the case of copper, a larger doping level, with a reduction of Cu^2+^ into Cu^+^, can be attained but necessitates a higher sintering temperature. The case of iron is also characterized by a high doping level that is achieved by the splitting of the cationic interstitial site away from the hexagonal axis [34]. The large ionic radius difference between Ca^2+^ and the doping 3d metal cations (Table 1) explains why either substitution or insertion mechanisms can appear in these systems.

## 2. Materials and Methods

### 2.1. Sol-Gel Elaboration of M-Doped BCP Samples

The sol-gel route previously proposed by the authors was used to synthesize undoped and *M*-doped series of BCP samples [15,16], with *M* = Mn^2+^, Fe^3+^, Co^2+^, Ni^2+^, Cu^2+^, and Zn^2+^. The sol-gel route developed by the authors has been used because of the well-documented ability and versatility of the sol-gel process to homogeneously incorporate a great variety of dopant. Briefly, to produce 2 g of undoped BCP powder, 4.7 g of Ca(NO_3_)_2_∙4H_2_O (Aldrich) and 0.84 g of P_2_O_5_ (Avocado Research Chemicals, Lancaster, UK) were dissolved in ethanol (anhydrous, >99.5%, Country Sigma-Aldrich, Saint Louis, MO, USA) under stirring and refluxed at 85 °C for 24 h. This solution was then maintained at 55 °C for 24 h to obtain a consistent white gel, then further heated at 80 °C for 10 h to obtain a white powder. Finally, the powder was heat treated for 15 h. Heat treatment was performed at 500 °C, 600 °C, 700 °C, 800 °C, 900 °C, 1000 °C, and 1100 °C. Selected series were also heat treated at 1150 °C and 1200 °C (Cu- and Mn-doped series). Required amounts of hydrated metal nitrate (Mn(NO_3_)_2_∙4H_2_O, Fe(NO_3_)_3_∙9H_2_O, Co(NO_3_)_2_∙6H_2_O, Ni(NO_3_)_2_∙6H_2_O, Cu(NO_3_)_2_∙3H_2_O, and Zn(NO_3_)_2_∙6H_2_O from Sigma-Aldrich (Saint-Louis, MO, USA) were added to the solution simultaneously with Ca(NO_3_)_2_∙4H_2_O (Sigma-Aldrich) to synthesize the corresponding *M*-doped series. To obtain series of samples with the same doping amount, a single batch was prepared for each series and used for all the subsequent heat treatments. Nominal compositions were calculated assuming the insertion of *M*^2+^ (Mn, Co, Ni, Cu, and Zn) or *M*^3+^ (Fe) cations at interstitial crystallographic sites (i.e., Ca/P = 1.67 invariably) in agreement with previous results for the Zn^2+^ insertion mechanism [14,15,16]. In the following, the samples are labeled “*xM*-T” with *x* = 0, 15, 25, 50, and 75 for samples with the targeted nominal Ca_10_(PO_4_)_6_(OH)_2_, Ca_10_*M*_0.15_(PO_4_)_6_(OH)_1.70_O_0.30_, Ca_10_*M*_0.25_(PO_4_)_6_(OH)_1.50_O_0.50_, Ca_10_*M*_0.50_(PO_4_)_6_(OH)_1.00_O_1.00_, and Ca_10_*M*_0.75_(PO_4_)_6_(OH)_0.50_O_1.50_ compositions for divalent cations, and Ca_10_Fe_0.15_(PO_4_)_6_(OH)_1.55_O_0.45_, Ca_10_Fe_0.25_(PO_4_)_6_(OH)_1.25_O_0.75_, Ca_10_Fe_0.50_(PO_4_)_6_(OH)_0.50_O_1.50_, and Ca_9.875_Fe_0.75_(PO_4_)_6_O_2_ compositions for the trivalent Fe^3+^ series. In this label, T indicates the sintering temperature (from 500 °C to 1200 °C). A total of 193 samples were prepared and characterized: nine undoped samples, 28 for each of the Fe-, Co-, Ni-, and Zn-doped series, and 36 for both the Mn- and Cu-doped series. Elemental analyses of the samples performed by ICP-AES confirmed the targeted nominal compositions (i.e., confirmed that the 3d metal cations incorporated in the solutions were well incorporated in the precipitates). The sample color is cation- and sintering temperature-dependent. 

### 2.2. X-ray Powder Diffraction (XRPD) and Rietveld Analyses

XRPD patterns were recorded on an X’Pert Pro diffractometer (Philips PANalytical, Almelo, The Netherlands), with θ-θ geometry, reflection mode, equipped with a X-Celerator solid detector and using Cu Kα radiation (λ = 1.54184 Å). XRPD patterns were recorded at room temperature in the interval 3° < 2θ < 120°, with a step size of ∆2θ = 0.0167° and a counting time of 200 s for each data value. A total counting time of about 200 min was used for each sample. An XRPD pattern was collected from pure NIST-standard LaB_6_ using the same experimental conditions in order to obtain the instrumental resolution function to improve peak profile fitting during Rietveld refinements and to extract microstructural parameters. The characterization of the mineral composition of the samples and of the lattice parameters of both HAp and β-TCP phases was systematically performed using Rietveld refinements, for all prepared samples. The location of the 3d metal doping cation was also systematically identified for both HAp and β-TCP phases.

Rietveld refinements were performed using the FullProf.2k program [37]. The procedure used (both data collection and refinement strategy) corresponds to the general guidelines for structure refinement using the Rietveld (whole-profile) method formulated by the International Union of Crystallography Commission on Powder Diffraction [38,39]. The Rietveld strategy was detailed in a previous related work [14]; the quality of refinements has been assessed using standard procedures and all reliability factors were below 5%.

### 2.3. X-ray Absorption Spectroscopy (XAS)

*M* K-edge Extended X-ray Absorption Fine Structure (EXAFS) spectra, simultaneously with the X-ray Absorption Near Edge Structure (XANES) part of the spectra, were collected for the samples from the 15*M*-T series (M = Zn, Cu, Ni, Co, Fe) and on various reference compounds (ZnO, CuO, NiO, Co(OH)_2_, Fe_2_O_3_, and Fe_3_O_4_) at the SuperXAS beam line at the SLS synchrotron (Villigen, Switzerland) in order to determine the electronic state as well as accurately describe the coordination spheres of *M* atoms. Samples from the 15*M*-T series were chosen because other series presented greater quantities of metal oxide impurities. The SLS synchrotron ran at 4.5–35 keV with an average current of 400 mA. The X-ray beam was obtained with a two-crystal Si(111) monochromator offering an energy resolution of ΔE/E = 2.0 × 10^−4^, necessary to resolve the XANES structure. The experiments were calibrated with metallic reference foils (K-edge 9659 eV for zinc, K-edge 8981 eV for copper, K-edge 8332 eV for nickel, K-edge 7709 eV for cobalt, and K-edge 7109 eV for iron). Experiments were performed at room temperature and atmospheric pressure. Spectra were collected in an energy range of 1000 eV, with energy steps varying from 0.5 eV (XANES part) to 2.0 eV (end of the EXAFS part) and 1 s dwell time per point. XAS spectra were obtained in fluorescence mode using Ge-solid-state detectors (13-element detector). The beam size was determined by a set of slits (200 μm × 500 μm). Data were processed using the Athena and Artemis programs from the IFFEFIT software package [40] by merging six successively recorded absorption spectra. Single scattering theory was used here. Following Lengeler–Eisenberg normalization, EXAFS oscillations were Fourier Transformed (FT) using a Hanning window between 3.0 and 9.0 Å^−1^. The χ(*k*) function was Fourier transformed using *k*^3^ weighting, and all shell-by-shell fitting was performed in *R*-space. Theoretical backscattering paths were calculated using successively ATOMS [41] and FEFF6 [42,43]. 

## 3. Results

### 3.1. Mineral Composition of the M-Doped BCP Samples

**Figure 1:**
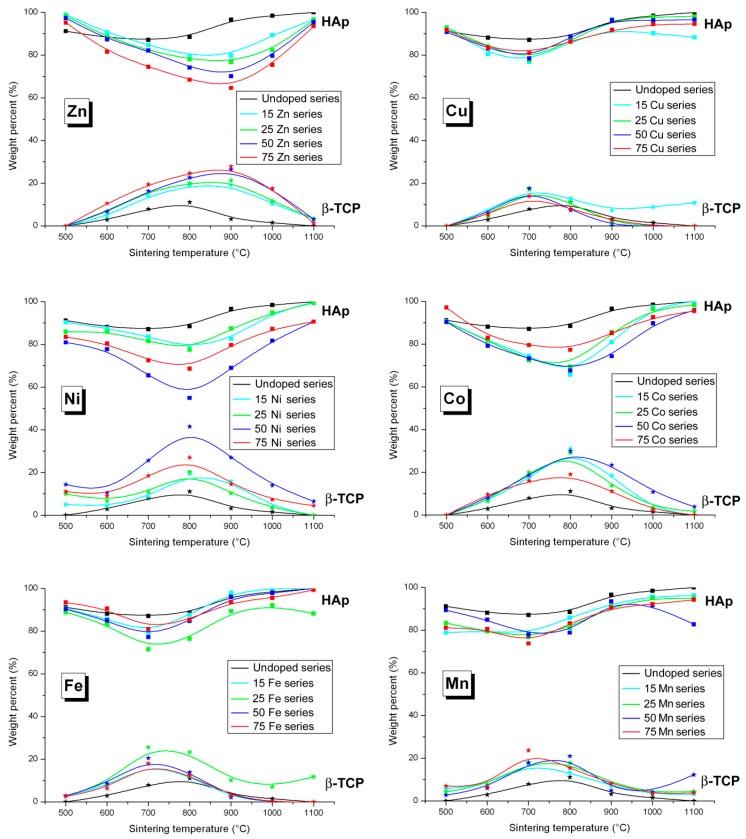
Weight percent of HAp (squares) and β-TCP (stars) obtained from Rietveld refinements for all the Zn-, Cu-, Ni-, Co-, Fe-, and Mn-doped samples: black lines for the undoped series, light blue lines for the 15*M*-T series, green lines for the 25*M*-T series, dark blue lines for the 50*M*-T series, and red lines for the 75*M*-T series. The lines are only visual guides.

Previous results on the Zn-doped BCP samples highlighted the temperature dependence of the HAP/β-TCP weight ratio [15]: the HAp phase is by far the main phase for a sintering temperature of 500 °C (almost single-phase samples); an increase in sintering temperature stabilizes the β-TCP phase with a maximum amount encountered at 800 °C for the undoped series and at 900 °C for the Zn-doped series; and finally a high sintering temperature again stabilizes the doped HAp structure. These previously published results are shown, together with the five other doped series, in Figure 1. It appears that the six series present generally similar behavior with some specific differences. The β-TCP structure is continuously stabilized for intermediate temperatures, but the maximum amount of β-TCP is metal-dependent as well as dependent on the sintering temperature for which the maximum amount of β-TCP is observed. Whereas a maximum of about 30 wt % is observed for the Co-series (similar to the Zn-series), it is higher for the Ni-series (close to 40 wt %) and lower for the Cu-, Fe-, and Mn-series (close to 20 wt %). On the other hand, the temperature producing the maximum quantity of β-TCP decreases from 900 °C for the Zn-series, to 800 °C for the Ni- and Co-series, and to 700 °C for the Cu-, Fe-, and Mn-series. It seems that the lower the amount of stabilized β-TCP, the lower the corresponding sintering temperature. It also appears from Figure 1 that some series are somewhat outside the general observations, indicating the necessity to carefully prepare and homogenize the pressed pellet before sintering. The 15Cu series shows a particularity, compared to the other *x*Cu series, with a persistency of about 10 wt % of β-TCP between 700 °C and 1100 °C. Such a particularity is also observed for the 25Fe series. Surprisingly, the 75Co series shows a continuously smaller β-TCP amount compared to the three other *x*Co series. To a lesser extent the 50Ni and 75Ni series seem to be reversed. Despite these few features, the stabilizing effect on the β-TCP phase at intermediate temperatures is clearly highlighted for all the studied samples.

**Figure 2:**
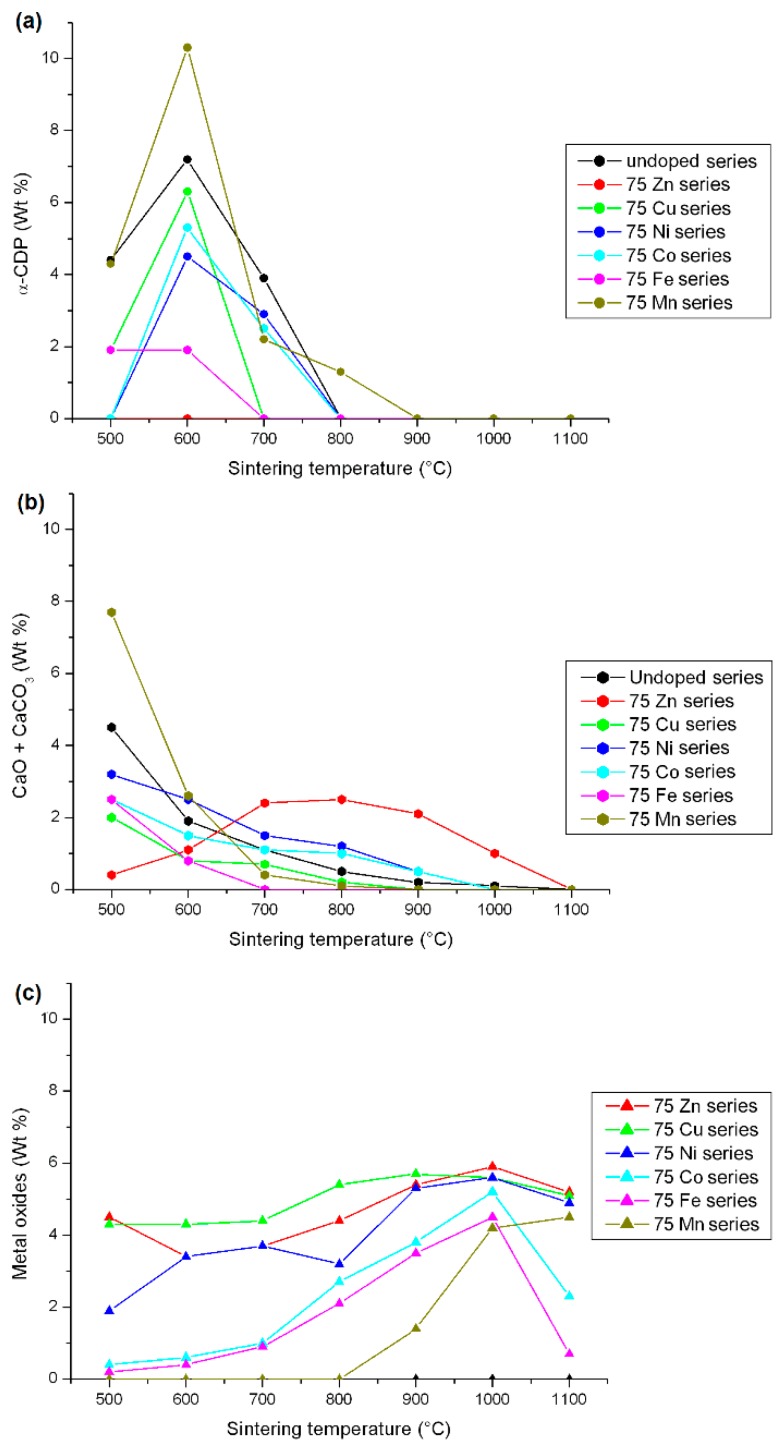
Weight percent of minor phases ((**a**) α -CDP; (**b**) sum of CaCO_3_ and CaO; (**c**) metal oxides) for the 75*M*-T samples: red lines for the 75Zn-T series with ZnO as metal oxide, green lines for the 75Cu-T series with CuO as metal oxide, dark blue lines for the 75Ni-T series with NiO as metal oxide, light blue lines for the 75Co-T series with Co_3_O_4_ and CoO as metal oxide, pink lines for the 75Fe-T series with Fe_2_O_3_ as metal oxide, and dark green lines for the 75Mn-T series with the sum of Mn_3_O_4_ and CaMn_2_O_4_ as metal oxide. The lines are only visual guides.

The weight amounts of the other minor phases are shown in Figure 2. Contrary to the Zn-doped case, α-CDP (alpha-dicalcium phosphate, composition Ca_2_P_2_O_7_) is generally observed for the lower sintering temperatures, with a maximum amount at 600 °C (Figure 2a). It is difficult to explain the presence of this calcium phosphate phase with a Ca/P ratio of only 1.0, far from the targeted Ca/P ratio of 1.67, simultaneously with about 5–10 wt % β-TCP with an already smaller 1.5 Ca/P ratio. However, the presence of α-CDP should not be attributed to the presence of the doping elements, since the undoped series already shows dicalcium phosphate for sintering temperatures between 500 °C and 700 °C. This third calcium phosphate phase completely disappears for sintering temperatures above 700 °C in general, above 800 °C for the Mn-series and already above 600 °C for the Fe- and the Cu-series. Concomitantly with the presence of α-CDP, CaCO_3_ and CaO are present for the lower sintering temperatures (Figure 2b). The presence of these phases counterbalances the low Ca/P ratio of α-CDP, as shown by the simultaneous higher amounts of α-CDP and (CaCO_3_ + CaO) for the 75Mn-series, closely followed by the undoped series. CaCO_3_ and CaO are summed in Figure 2 because these two phases correspond to Ca^2+^ cations which are not combined with phosphate anions, and because CaCO_3_ transforms into CaO at around 800 °C (calcite decarbonation). Except for the 75Zn-series, the (CaCO_3_ + CaO) amount decreases below 1 wt % after 900 °C, when the targeted HAp phase becomes the main phase with almost 90 wt %. The behavior of the Zn-doped system is different from the others, with a very low amount of (CaCO_3_ + CaO) at 500 °C (linked to the absence of α-CDP), followed by an increase up to about 3 wt % at 900 °C, before the complete disappearance of CaO at 1100 °C. In the Zn-doped system without the formation of α-CDP, the presence of calcium-rich phases is directly correlated with the weight amount of β-TCP: the (CaCO_3_ + CaO) sum is close to zero for the lower and the higher sintering temperatures when the Zn-doped samples are mainly composed of the HAp phase. The fact that (CaCO_3_ + CaO) are present in BCP to counterbalance the presence of a calcium phosphate phase with a Ca/P ratio smaller than the targeted 1.67 value is strengthened in the Cu- and Fe-doped BCP systems, which show smaller (CaCO_3_ + CaO) amounts, in agreement with the larger amount of HAp phase whatever the sintering temperature.

**Figure 3:**
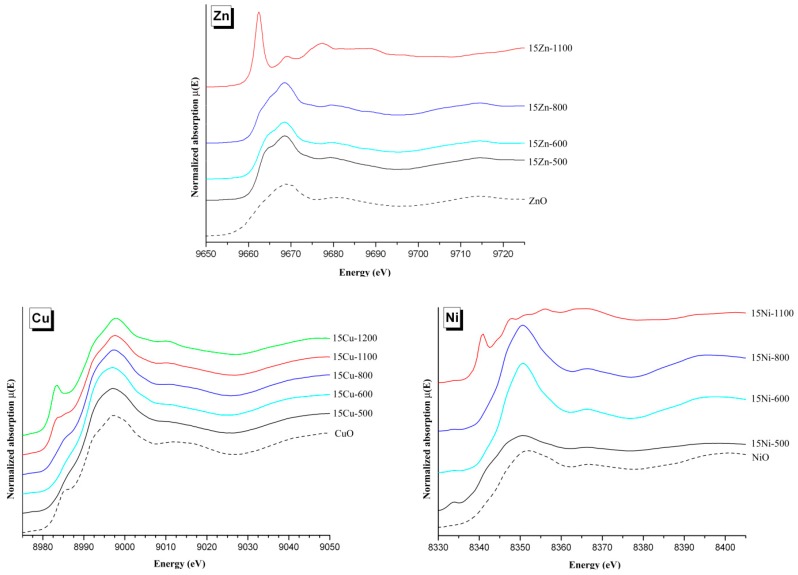

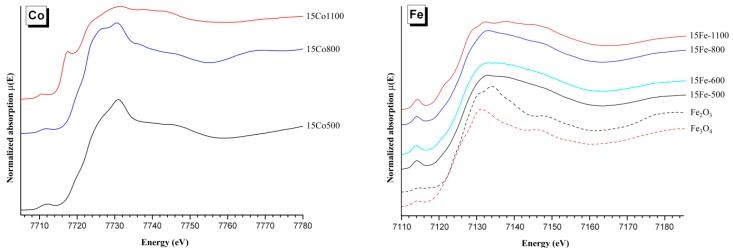
Normalized XANES spectra of the *M* K-edge for samples from the 15*M*-T series and selected reference materials (*M* = Zn, Cu, Ni, Co, and Fe).

Figure 2c illustrates the quantity of metal oxide phases present in the 75*M* series, i.e., the quantity of doped elements not incorporated into either the HAp phase or the β-TCP phase (incorporation into the minor α-CDP phase was not considered here). Two behaviors can be identified from Figure 2c: (1) the 75Zn- and 75Cu-series show a relatively constant amount of ZnO and CuO, about 5 wt %, whatever the sintering temperature; and (2) the 75Co- and 75Fe-series show an increase in Co_3_O_4_ and Fe_2_O_3_ amounts from almost zero at 500 °C to about 5 wt % at 1100 °C. The 75Ni-series is intermediate, and the last 75Mn-series belongs to the second case with the appearance of metal oxides (Mn_3_O_4_ and CaMn_2_O_4_) from 900 °C only. Two locations have to be kept in mind for the doping elements that are not included in these undesirable metal oxides: either incorporated into one or both HAp and β-TCP phases or engaged in an amorphous phase that could be the HAp surface (not detectable by XRPD). The presence of a doping element in an amorphous phase was highlighted in the Zn-doping case [14,15,16] and the Fe-doping case [34], in particular for lower sintering temperatures. X-ray absorption spectroscopy has indicated that PXRD-undetectable Zn^2+^ cations are certainly in tetrahedral coordination located in an amorphous shell at the HAp crystal surface. The observed metal oxides also indicate a possible change in the oxidation state of doping element oxidation. Whereas no oxidation was observed for the divalent cation Zn^2+^ (with ZnO formation), Cu^2+^ (with CuO formation), Ni^2+^ (with NiO formation), or for the trivalent cation Fe^3+^ (with Fe_2_O_3_ formation), modifications were in evidence for both the cobalt and manganese systems. Divalent Co^2+^ and Mn^2+^ were introduced during the syntheses (using Co(NO_3_)_2_∙6H_2_O and Mn(NO_3_)_2_∙4H_2_O, respectively) and spinel-type materials with trivalent cations were observed: Co_3_O_4_ containing both Co^2+^ and Co^3+^, Mn_3_O_4_ containing both Mn^2+^ and Mn^3+^ and CaMn_2_O_4_ containing Co^3+^. It is also important to bear in mind a possible change in the oxidation state of the doping element when incorporated into one of the two main phases of the BCP samples; careful analyses of the XANES part of the SAX spectra (Figure 3) will allow such oxidation or reduction of the doping cation to be highlighted. 

### 3.2. M Incorporation into the β-TCP Structure

**Figure 4:**
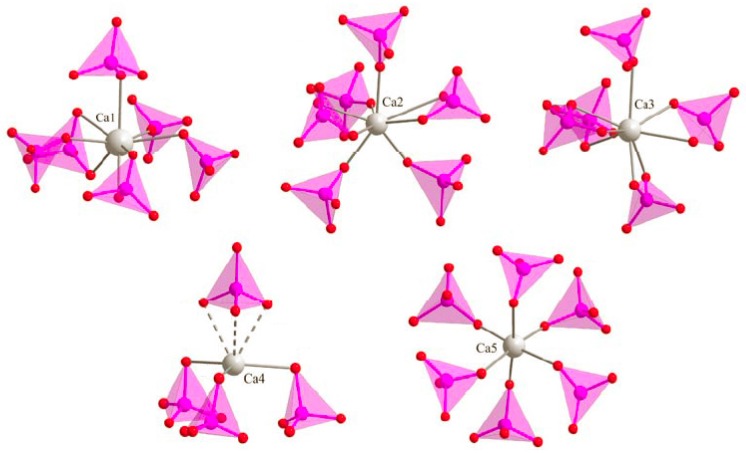
Local environment of the five calcium crystallographic sites in the β-TCP structure (**gray** spheres represent the calcium atoms and **pink** tetrahedra represent the phosphate groups).

**Figure 5:**
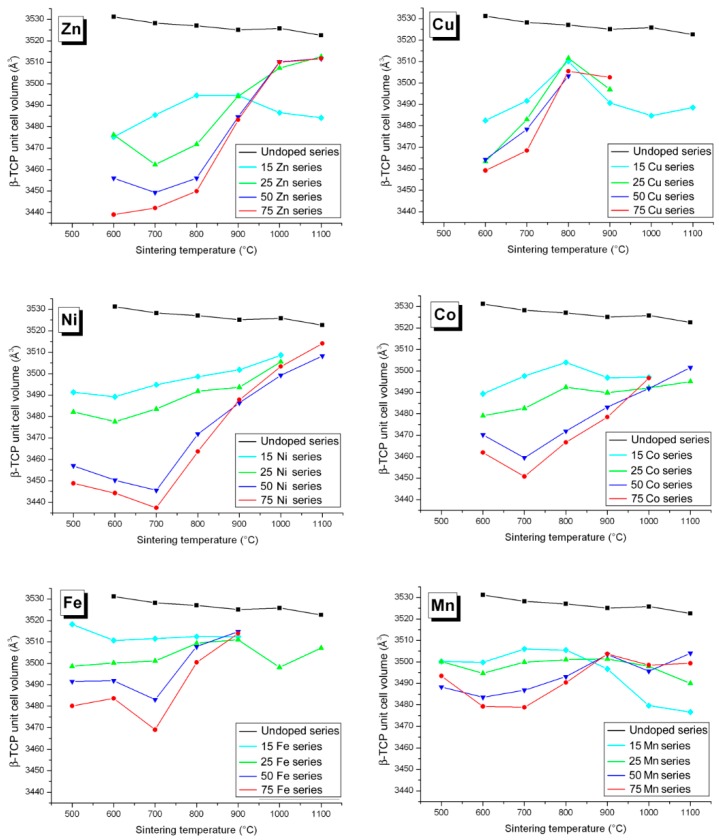
Variation in the unit cell volume of the β-TCP phase from all Zn-, Cu-, Ni-, Co-, Fe-, and Mn-doped samples: black lines for the undoped series, light blue lines for the 15*M*-T series, green lines for the 25*M*-T series, dark blue lines for the 50*M*-T series, and red lines for the 75*M*-T series. The lines are only visual guides.

**Figure 6:**
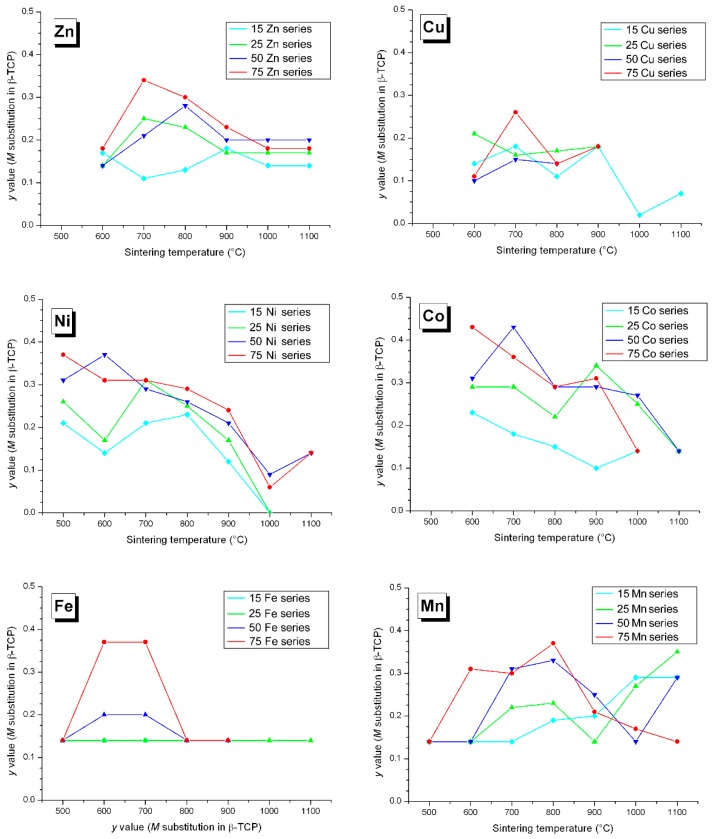
Variation in the *y* refined substituted *M* amount in the β-TCP structure, considering the Ca_3−*y*_*M_y_*(PO_4_)_2_ composition, for all Zn-, Cu-, Ni-, Co-, Fe-, and Mn-doped samples: light blue lines for the 15*M*-T series, green lines for the 25*M*-T series, dark blue lines for the 50*M*-T series, and red lines for the 75*M*-T series. The lines are only visual guides.

Previous studies of Zn-doping insertion in BCP have shown a substitution mechanism for β-TCP leading to the doped chemical composition Ca_3−*y*_*M_y_*(PO_4_)_2_ when considering divalent cations, and Ca_3−3*y/2*_Fe*_y_*(PO_4_)_2_ for trivalent Fe^3+^. Calcium substitution was evidenced by a simultaneous decrease in both the *a* and *c* hexagonal lattice parameters, due to the smaller cationic radius of Zn^2+^ compared to Ca^2+^ (Table 1). The two six-fold coordinated calcium crystallographic sites Ca4 (deformed trigonal prism) and Ca5 (octahedron) are concerned by zinc substitution (Figure 4). The three other eight-fold coordinated Ca1, Ca2, and Ca3 calcium sites are too large to accept small Zn^2+^ cations [36]. The Zn case, shown in Figure 5, highlights that Zn substitution is predominant at 700 °C–800 °C when the β-TCP phase is the more abundant (about 20 wt %; Figure 1). The β-TCP unit cell volume decrease is more pronounced when the Zn amount in the overall BCP composition increases (from 1.2% for 15Zn-700 to 1.8% for 25Zn-700 to 2.2% for 50Zn-700 and to 2.4% for 75Zn-700), indicating a higher *y* value for the Zn-doped Ca_3−*y*_Zn*_y_*(PO_4_)_2_ phase. Refined *y* values (shown in Figure 6), although moderately accurate due to the numerous crystallographic site positions in the β-TCP structure combined with small weight amounts of the phase, are more or less correlated with the unit cell volume variations. An average *y* value of 0.2, corresponding to the Ca_2.8_Zn_0.2_(PO_4_)_2_ phase, is found for all the Zn-doped BCP samples, with a maximum *y* value of 0.35 (Ca_2.65_Zn_0.35_(PO_4_)_2_) found for the 75Zn-700 sample. The six *M*-doped systems behave relatively similarly in terms of β-TCP unit cell volume variations (Figure 5) with respect to the refined *y* values (Figure 6). The six incorporated cations present quite similar cationic radii in six-fold coordination (Table 1). As can be seen in Figure 5, the maximum unit cell volume contraction is observed for Ni-doping (the smallest doping cation, with a radius of 0.69 Å), at about 2.6% for 75Ni-700. On the contrary, a smaller unit cell volume contraction is observed for the Mn-doping system (with an average and quite constant contraction of 0.9%), corresponding to the larger doping cation with a radius of 0.83 Å (HS configuration). As observed in the Zn-doping system, and except for the Mn-doping system, all the other systems show a decrease in the unit cell volume contraction with an increase in the sintering temperature. Such an observation indicates the transfer of the doping element from the β-TCP to the HAp structure when the latter becomes overwhelmingly the major phase. An increase in sintering temperature (above 900 °C) stabilizes the *M*-doped HAp phase at the expense of the *M*-doped β-TCP phase. The β-TCP phase decreases in weight amount and also in doping level for the higher sintering temperatures. The decrease in the doping level with temperature is illustrated in Figure 6. This is not very clear for all doping systems, but a general overview gives an average *y* value of 0.2, which decreases from ~0.3 at 500 °C to ~0.1 at 1000 °C; this is especially true for the Ni-doping system. This overall tendency seems valid for all the systems except for Mn-doping, for which the unit volume contraction, as well as the *y* refined value, remained quite constant at all sintering temperatures. 

### 3.3. M Incorporation into the HAp Structure

**Figure 7:**
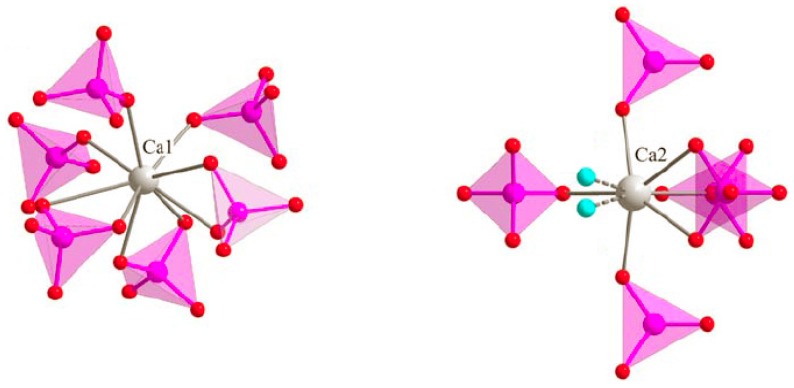
Local environment of the two calcium crystallographic sites in the HAp structure (**gray** spheres represent the calcium atoms, **pink** tetrahedra represent the phosphate groups, and **light blue** spheres represent the half-occupied hydroxyl position).

**Figure 8:**
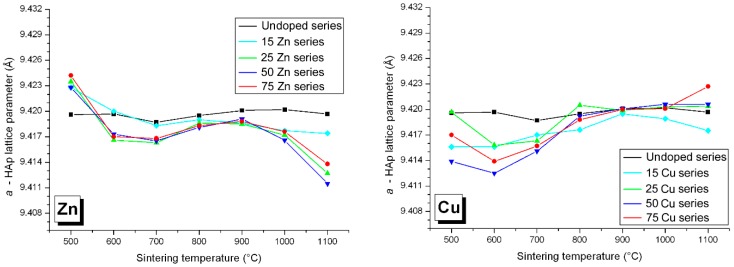

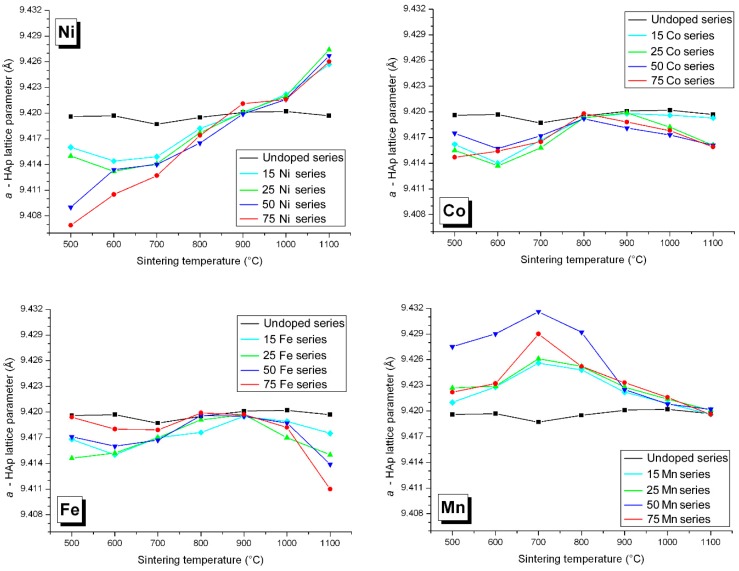
Variation in the basal *a* lattice parameter of the HAp phase from all Zn-, Cu-, Ni-, Co-, Fe-, and Mn-doped samples: black lines for the undoped series, light blue lines for the 15*M*-T series, green lines for the 25*M*-T series, dark blue lines for the 50*M*-T series, and red lines for the 75*M*-T series. The lines are only visual guides.

**Figure 9:**
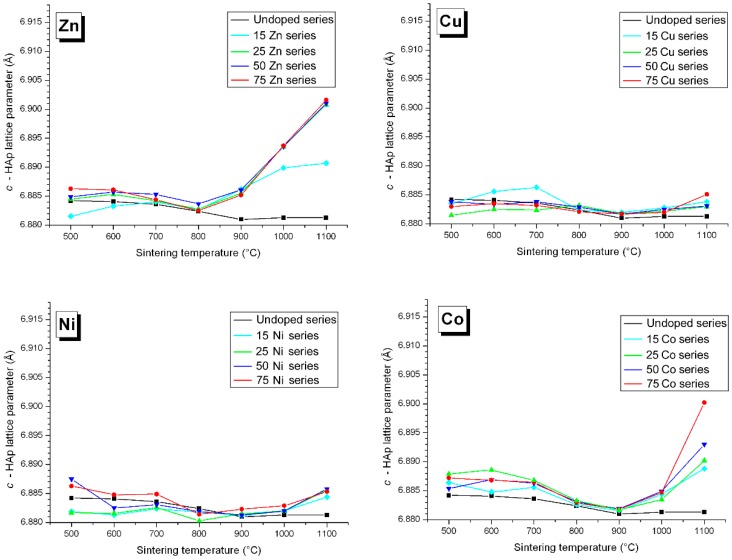

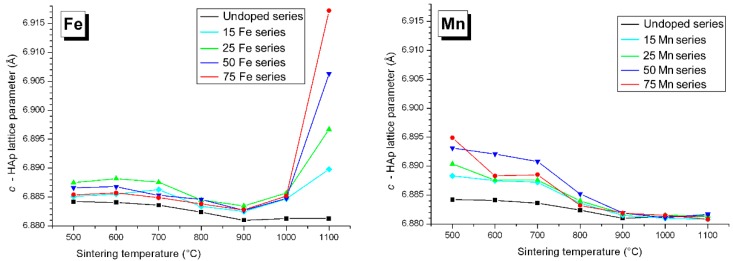
Variation in the hexagonal *c* axis of the HAp phase from all Zn-, Cu-, Ni-, Co-, Fe-, and Mn-doped samples: black lines for the undoped series, light blue lines for the 15*M*-T series, green lines for the 25*M*-T series, dark blue lines for the 50*M*-T series, and red lines for the 75*M*-T series. The lines are only visual guides.

**Figure 10:**
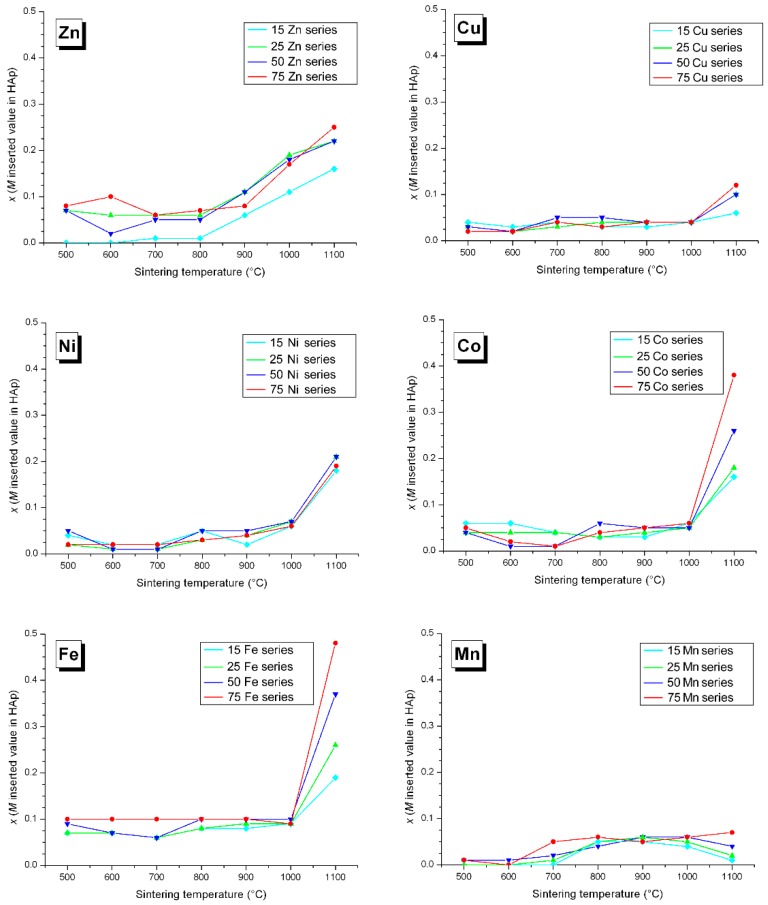
Variation in the *x* refined *M* amount inserted into the HAp structure, considering the Ca_10_*M_x_*(PO_4_)_6_(OH)_2−2*x*_O_2*x*_ composition, for all the Zn-, Cu-, Ni-, Co-, Fe-, and Mn-doped samples: light blue lines for the 15*M*-T series, green lines for the 25*M*-T series, dark blue lines for the 50*M*-T series, and red lines for the 75*M*-T series. The lines are only visual guides.

We have shown in previous Zn-doping studies that Zn^2+^ incorporation into the HAp structure is not effected by substitution at the Ca1 and/or Ca2 calcium sites (Figure 7, respectively nine-fold and seven-fold coordinated [44]), but by insertion along the hexagonal channel [14,15,16]. The Zn partial occupancy of the 2*b* crystallographic site was proven using a combination of X-ray and neutron powder diffraction performed on the same samples. Independent Rietveld analyses indicated the same occupancies when considering Zn^2+^ in the 2*b* position [14]. The linear O–Zn–O entity formed, located along the hexagonal channel, was characterized by EXAFS analysis, with the first description of a two-fold coordination for Zn^2+^ [16]. The interstitial mechanism was evidenced by the anisotropic unit cell variation: a decrease of the basal *a* lattice parameter combined with an increase of the hexagonal *c* lattice parameter. Figure 8 and Figure 9 illustrate that Zn^2+^ insertion into the HAp structure is mainly realized for a sintering temperature above 900 °C, to form the Ca_10_Zn*_x_*(PO_4_)_6_(OH)_2−2*x*_O_2*x*_ phase with *x* < 0.25 (the maximum *x* value being obtained at 1100 °C only, Figure 10). Cationic insertion is counterbalanced by proton departure (leading to hydroxide to oxide substitution) because no calcium deficiency was evidenced [15]. A comparison with the five other investigated cations in Figure 8 and Figure 9 shows different behaviors: (1) Fe^3+^ is the only other cation with an anisotropic unit cell variation; (2) Co^2+^ induces an increase in the hexagonal *c* lattice parameter without variation in the basal *a* lattice parameter; (3) Ni^2+^ induces an increase in the basal *a* lattice parameter without variation in the hexagonal *c* lattice parameter; (4) Cu^2+^ incorporation does not show significant lattice parameter variations (or only a weak basal *a* lattice parameter contraction for lower sintering temperatures); (5) Mn^2+^ incorporation induces an increase in both *a* and *c* lattice parameters for lower sintering temperatures. Thus it appears that the 3d metal doping mechanism of the HAp phase is not as simple as that described for Zn^2+^. Figure 10 shows the *x* refined value obtained from the occupancy parameter for the cation located in the interstitial 2*b* Wyckoff position. Despite all the different HAp unit cell volume variations, an insertion into the interstitial site appears for a large proportion of the investigated cations; only Mn^2+^ seems not to be located at the 2*b* Wyckoff site. Attempts at calcium substitution in both Ca1 and Ca2 sites were systematically tested for each cation and each sintering temperature, and invariably a refined substitution level close to zero was obtained. It should be noted that when passing from Zn^2+^ to Mn^2+^, the electronic contrast with a Ca^2+^ cation is progressively weaker and consequently the 3d metal to calcium substitution is less and less detectable by X-ray diffraction. When considering *M^n^*^+^ insertion into the 2*b* Wyckoff site, the maximum *x* value of 0.25 reported for Zn^2+^ [15] is also valid for Ni^2+^ and Co^2+^. The case of Fe^3+^ shows that higher inserted amounts are possible, with *x* ~ 0.5 obtained at 1100 °C for the 75Fe-series. Cu^2+^ evinces a lower insertion level with only *x* ~ 0.1 at 1100 °C for the 75Cu-series, while the Mn-series seems to indicate even lower *x* values.

**Figure 11:**
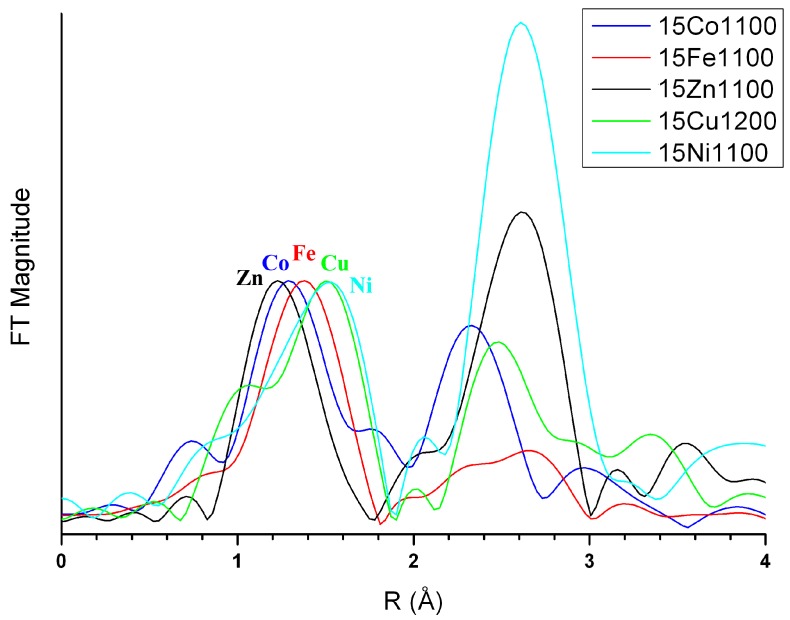
*k*^3^-weighted amplitude of the Fourier transform, uncorrected for phase shift, for 15Zn-1100 (black line), 15Cu-1200 (green line), 15Ni-1100 (light blue line), 15Co-1100 (blue line), and 15Fe-1100 (red line).

The incorporation of metallic cations into the HAp phase is achieved by their insertion into its hexagonal channel, leading to low coordination of the doping element. Processing the EXAFS data confirms these observations, with short *M*–O distances. Figure 11 highlights the first coordination shell. In each case, small coordination, associated with short interatomic distance (uncorrected from phase shift in Figure 11), has been calculated.

## 4. Discussion

The results obtained indicate that, overall, 3d metal incorporation in BCP is common for the six studied cations. The temperature-dependent mechanism, with incorporation into β-TCP for intermediate temperatures involving a substitution mechanism, followed by incorporation into HAp for higher temperatures at an interstitial crystallographic site, is valid whatever the 3d metal cation. Nevertheless, careful observations of the doping mechanism of the HAp structure show specific characteristics in comparison with the Zn doping mechanism; they are presented and discussed here.

### 4.1. Iron and Cobalt Doping Mechanism

**Figure 12:**
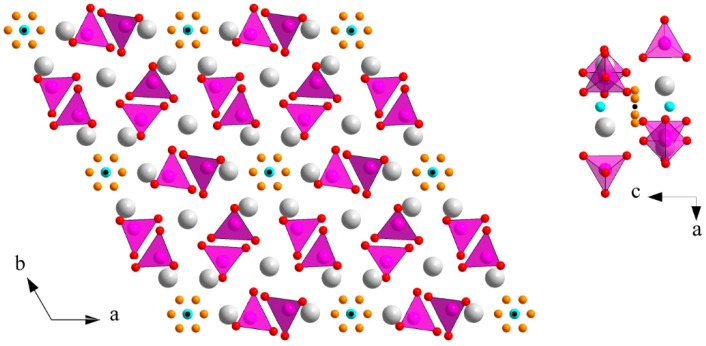
Structural representations showing the six-fold split characteristic of the Fe^3+^-doping mechanism: projection in the basal (*a*,*b*) plane and perpendicular to the hexagonal *c* axis (right). **Gray** spheres represent the calcium atoms, **pink** tetrahedra represent the phosphate groups, **light blue** spheres represent the hydroxyl position, small **black** spheres represent the central *M* positions for Zn^2+^, Cu^2+^, and Ni^2+^, and orange spheres represent the *M* split position from the center of the hexagonal channel for Fe^3+^ and Co^2+^.

The iron series variations are very similar to the zinc case, with an easier stabilization of the Fe:HAp phase compared to the Zn:HAp doped phase, as illustrated in Figure 1: the amount of β-TCP phase is smaller for the Fe-BCP series and already considerably decreases at 900 °C (the stabilization temperature of β-TCP for the Zn-BCP series). It also appears that the β-TCP phase accepts less iron-to-calcium substitution compared to zinc. This is observable both in Figure 5, with a lower decrease in the β-TCP unit cell volume in presence of iron despite a smaller ionic radius (0.64 Å and 0.78 Å for Fe^3+^ respectively six- and eight-fold coordinated, compared to 0.74 Å and 0.90 Å for Zn^2+^), and in Figure 6, with the *y* refined substitution value generally below 0.2. This lower affinity of iron for the β-TCP phase is coupled with a greater affinity with the HAp structure. Figure 8 shows approximately the same basal *a* lattice contraction when inserting either Zn^2+^ or Fe^3+^, but Figure 9 clearly shows a greater increase in the hexagonal *c* lattice parameter in the case of Fe^3+^ insertion. These observations are correlated with a higher inserted level (Figure 10) in the case of iron: Ca_10_*M^n^*^+^*_x_*(PO_4_)_6_(OH)_2−*nx*_O*_nx_* with *x_max_* ~ 0.25 for Zn^2+^ and ~0.50 for Fe^3+^. Nevertheless, high inserted iron levels were not evidenced with the 3d metal cation located in the 2*b* Wyckoff position. In this position, the refined amount of Fe^3+^ was always low, with *x* < 0.2, but with thermal isotropic factors strangely and unusually high. The Rietveld refinements of the 50Fe-T and 75Fe-T samples for T = 1100 °C were considerably improved by considering Fe^3+^ cations which were not located exactly in the 2*b* Wyckoff position but shifted outside the hexagonal axis. Instead of using the fixed (0,0,0) atomic coordinates for Fe^3+^, the 12*i* (*x*,0,0) Wyckoff site was used with the refinement of the *x* coordinate at a value close to 0.12. This enabled the Rietveld refinement to be improved, and the refined inserted amount of Fe^3+^ to be increased, in agreement with the large increase in the hexagonal *c* lattice parameter. The six-fold split position of the Fe^3+^ cation allows a considerable increase in the amount of Fe^3+^ inserted into the HAp structure at 1100 °C (Figure 10), and is concomitant with the disappearance of the iron Fe_2_O_3_ oxide impurity from the samples between 1000 °C and 1100 °C (Figure 3, bottom). A crystallographic description of our samples indicates that the iron cation is located at the 2*b* Wyckoff site for the lower sintering temperatures, and is shifted to the 12*i* Wyckoff site from 1000 °C upwards. This shift implies an increase in the Fe^3+^ coordination from a linear two-fold coordination (two oxygen atoms from the hydroxyl crystallographic site) to a triangular three-fold coordination (two oxygen atoms from hydroxyl sites and a third oxygen atom from the phosphate group). Iron Mossbauer spectroscopy [34] did not show such a sharp transition at above 1000 °C from the two-fold to the three-fold coordination, but a gradual evolution from 500 °C to 1100 °C, with the simultaneous presence of both coordinations in an evolving ratio. Such a shift from the center of the hexagonal channel has already been described in the case of Co-doped belovite, the strontium apatite Sr_10_(PO_4_)_6_(OH)_2_ analogue [45]. The authors indicated the chemical Sr_10_Co_0.4_(PO_4_)_6_(OH)_0.8_O composition and used a high annealing temperature of 1400 °C. They refined a 0.6 Å shift of Co^2+^ from the center of the hexagonal channels of the belovite structure, compared to our refined shift of 1.1 Å from the hexagonal channel of the hydroxyapatite structure. The large shift of Fe^3+^ in HAp clearly evidenced the three-fold coordination for the inserted 3d cation, with three almost equivalent Fe–O distances of around 1.8–1.9 Å, contrary to the insertion of Co^2+^ into belovite, which had two short Co–O distances estimated at around 1.7 Å, and two larger Co–O distances at 2.6–2.8 Å. The triangular three-fold coordination for Co^2+^ has already been described in the K_2_CoO_2_ compound [46]. Figure 12 illustrates the six-fold split of a Fe^3+^ cation leading to the three-fold coordination by an additional bonding with one phosphate group. Application of 3d cation splitting also enabled an improvement in the Rietveld refinements of the Co-doped series. For the highest doping level, heat treated at 1100 °C (samples 50Co-1100 and 75Co-1100), a refined shift of 0.9 Å from the center of the hexagonal channel was obtained, with a maximum inserted Co^2+^ amount corresponding to the composition Ca_10_Co_0.36_(PO_4_)_6_(OH)_1.28_O_0.72_. For the Co-BCP samples it is quite surprising to observe a variation in the cobalt oxide impurity content above 900 °C. Excess cobalt atoms (i.e., not included in one of the calcium phosphate phases) were found to form the spinel Co_3_O_4_ already at 500 °C for the 75Co-series, implying partial Co^2+^ to Co^3+^ oxidation (in agreement with thermal treatments in the air). From 1000 °C the weight amount of Co_3_O_4_ decreases and the divalent CoO impurity appears. This should indicate either that trivalent Co^3+^ cations extracted from the spinel compound are inserted into the HAp structure and divalent Co^2+^ cations are now located in the CoO metal oxide, or that trivalent Co^3+^ cations are reduced to divalent Co^2+^ which are inserted the HAp structure and also located in the CoO metal oxide. XANES spectra (Figure 3) enable us to discriminate oxidation states, and also to give information on site environments. Normalized Co K-edge XANES spectra are composed of three distinct edge features [47,48]. A pre-edge at about 7710 eV represents an electric dipole-forbidden (1s→3d) transition in an ideal octahedral environment; its presence indicates a non-centrosymmetric site for cobalt cations. The main edge at about 7730 eV and its shoulder at about 7720 eV correspond to the electric dipole-allowed (1s→4p) transition. The main edges, which are sensitive to the metal oxidation state, are close to 7726 eV for the three XANES spectra shown in Figure 3 for the Co-doped sample. This indicates quite the same Co^2+^/Co^3+^ ratio for the Co-doped samples, whatever the sintering temperature. The sharp and intense shoulder (7716.5 eV) is characteristic of a cobalt cation inserted into the HAp hexagonal channel: such sharp shoulders were observed, more or less, for all the doped samples annealed at 1100 °C (Figure 3). On the other hand, variations were observed for the pre-edge and the shoulder. A pre-edge is present for the three spectra, but shifts from 7712 eV (for 15Co500 and 15Co800 samples) to 7710 eV for the 15Co1100 sample). A pre-edge below 1000 °C corresponds to cobalt cations located in a tetrahedral environment from the spinel Co_3_O_4_ structure, whereas a pre-edge above 1000 °C (where the spinel has disappeared) indicates a non-centrosymmetric environment for Co^3+^ inserted into the HAp structure, which is in favor of the three-fold coordination (in the six-fold split position). Co K-edge spectra indicate Co^3+^ insertion into the HAp hexagonal channel with a trigonal three-fold coordination, in a similar manner to the trivalent Fe^3+^ cations.

### 4.2. Nickel Doping Mechanism

Among the three remaining systems, the Ni-doped BCP series behave very similarly to the Zn-doped series. Indeed, the HAp/β-TCP weight ratio variations for the Ni-series are not very different from those of the Zn-series (Figure 1); the Ni-doped and Zn-doped β-TCP phases are similar in unit cell volume and 3d cation substitution (Figure 6 and Figure 7), and the maximum 3d cation amount inserted into the HAp structure is about *x* ~ 0.25 (Ca_10_*M*_0.25_(PO_4_)_6_(OH)_1.5_O_0.5_ with M = Zn, Ni) for both systems (Figure 10). This can be explained by the previously reported linear two-fold coordination for Ni^2+^ cations in the belovite structure (situation identical to the description of Zn^2+^ insertion in hydroxyapatite) and in the compound *A*_2_NiO_2_ with *A* = K and Rb [49,50]. The main difference between the two systems resides in the HAp lattice parameter temperature dependence. The anisotropic variation in the hexagonal lattice parameters observed for Zn-doped HAp is not valid for the Ni-doped HAp phase (Figure 8 and Figure 9). The basal *a* lattice parameter of a Ni-doped HAp unit cell is smaller than for the undoped reference HAp series for the lower sintering temperatures, and it continuously increases with the sintering temperature until it exceeds the undoped HAp series above 1000 °C. The low temperature contraction observed for the *a* lattice parameter in the presence of nickel may reflect the existence of nickel-to-calcium substitution between 500 °C and 700 °C, although this was not confirmed by Rietveld refinements (either because the electronic scattering contrast is too low or because calcium substitution is accompanied by calcium site deficiency leading to a similar electronic contrast). On the other hand, the hexagonal *c* lattice parameter variations of the Ni-doped series are not really different from those of the undoped series. The absence of *c* lattice parameter expansion may be due to the somewhat shorter Ni–O distance compared to the Zn–O distance when considering linear two-fold coordination; respectively 1.64 Å [45] and 1.72 Å [16]. It appears from these last observations that the Ni-doping mechanism of the HAp structure implies a calcium substitution mechanism at 500–700 °C, which is replaced by an interstitial 2*b* Wyckoff site mechanism above 1000 °C. 3d metal incorporation into the BCP mechanism seems to be even more complicated than previously described for a Zn-doped system: calcium substitution in the HAp structure, potentially with calcium deficiency, counterbalances interstitial insertion. A general Ni-doped HAp formulae can be written: Ca_10−*x*−*y*_Ni*_x_*_+*z*_(PO_4_)_6_(OH)_2+2*y*−2*z*_O_2*y*−2*z*_ (with *y* < *z*) where *x*, *y*, and *z* represent calcium substitution, calcium site deficiency, and hexagonal channel insertion, respectively. The previously described Zn-doping mechanism is equivalent, with *x* and *y* close to zero.

### 4.3. Copper and Manganese Doping Mechanisms

**Figure 13:**
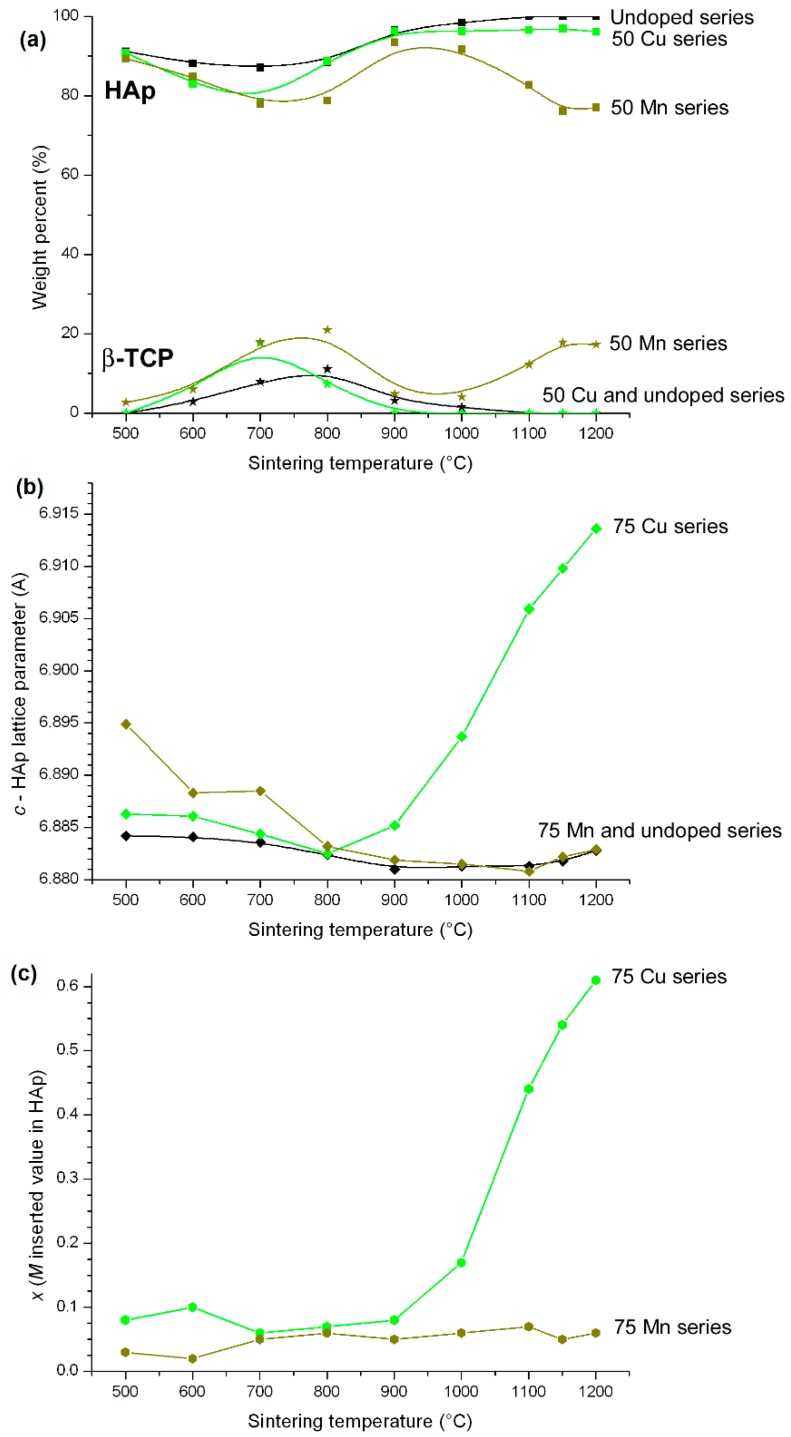
Different behaviors of the 75Cu-T series (green lines) and 75Mn-T series (dark green lines) when heat-treated up to 1200 °C ((**a**) weight percent of HAp and β-TCP obtained from Rietveld refinements; (**b**) variation in the hexagonal *c* axis of the HAp phase; (**c**) variation in the *x* refined *M* amount inserted into the HAp structure considering the Ca_10_*M_x_*(PO_4_)_6_(OH)_2−2*x*_O_2*x*_ composition). An undoped series (black lines) is shown for comparison. The lines are only visual guides.

The last two cases, Cu-doped and Mn-doped BCP systems, behave very differently to the Zn-doped case. First of all, these systems are less stabilizing with respect to the intermediate *M*-doped β-TCP phase for sintering temperatures between 600 °C and 800 °C (less than 20 wt % for the maximum β-TCP weigh amount at 700 °C). In particular, the *M*^2+^ amount inserted into HAp remains very low, whatever the sintering temperature (Figure 10). Attempts were made to increase the effect of sintering temperature up to 1200 °C. As shown in Figure 13, the two systems behave very differently at 1200 °C. An increase in the sintering temperature for the Mn system did not enable an increase in the very low amount of Mn^2+^ cation inserted into the HAp structure (Figure 13, bottom), correlated with the same hexagonal *c* lattice parameters for both the undoped and the Mn-doped series (Figure 13, middle). In addition, an increase in the sintering temperature destabilized the HAp phase at the expense of the β-TCP phase, with about 20 wt % at 1200 °C (Figure 13, top). On the other hand, an increase in the sintering temperature strongly affects the insertion of copper into the HAp structure. When passing from 900 °C to 1200 °C, Cu-doped HAp is still stabilized with almost no β-TCP phase present in the BCP samples, and the hexagonal *c* lattice parameter increases considerably up to a value similar to that observed in the Fe-doped system, corresponding to an inserted copper amount of 0.6 with a Ca_10_Cu_0.6_(PO_4_)_3_(OH)_0.8_O_1.2_ doped hydroxyapatite composition. In fact the latter chemical formula, assuming the insertion of Cu^2+^, is not correct, because the reduction of a large part of the Cu^2+^ to Cu^+^ was evidenced by the XANES experiment and confirmed by XPS measurements. This surprising high-temperature copper reduction will be detailed elsewhere. The Cu^2+^ to Cu^+^ reduction is evidenced in Figure 3 by the shift of the Cu K-edge shoulder from 8985 eV for the 15Cu800 sample to 8982 eV (sharper signal, characteristic of the HAp insertion mechanism) for the 15Cu1200 sample. An explanation for the difference in behavior between the Cu and Mn systems was obtained using in situ high-temperature powder X-ray diffraction experiments (from room temperature up to 1200 °C). Above 1100 °C the α-TCP phase—the high temperature stable TCP polymorph—is stabilized at the expense of β-TCP of course, but also at the expense of HAp. This is the reason why we generally halted our studies at 1100 °C. Upon cooling, the Mn-doped α-TCP phase formed at high temperature transforms into its Mn-doped β-TCP polymorph, and no Mn^2+^ insertion into HAp occurs. The copper case is different: temperatures above 1100 °C stabilized the Cu-doped α-TCP polymorph and also the isostructural Cu_3_(PO_4_)_2_ phase, which transform into a Cu-inserted HAp phase (and not into the Cu-doped β-TCP polymorph). The Mn-doped system remains mysterious, with apparently no Mn^2+^ incorporation into the HAp phase and relatively low calcium substitution in the β-TCP phase. However, HAp lattice parameters, particularly for the lower sintering temperatures, are higher than those from the undoped series. This seems to indicate the incorporation of Mn^2+^ into the HAp structure, but by an as yet unresolved mechanism; 2*b* Wyckoff site insertion was not evidenced during Rietveld refinement, and calcium substitution should induce lattice parameter shortening, contrarily to the observed increase. For now, difference Fourier maps have not enabled us to locate a new crystallographic site for Mn^2+^ in the HAp structure. Low manganese incorporation into the HAp structure has already been reported [33,51], generally with a calcium substitution mechanism (with a preference for the Ca1 site) [52].

## 5. Conclusions

We present here a complete description of the first-row transition metal doping mechanism in BCP. The previously described temperature-dependent mechanism for Zn^2+^ is valid for the whole investigated series from Mn^2+^ to Zn^2+^. Intermediate sintering temperatures (between 600 °C and 900 °C) stabilize the metal-doped M:β-TCP phase, with general formulae Ca_3−*x*_M*_x_*(PO_4_)_2_ obtained by calcium substitution at the two Ca4 and Ca5 crystallographic sites. This doped β-TCP phase, with a mean x value close to 0.2, composes about 20 wt % of the BCP samples (up to 40 wt %, depending on the doping metallic cation and the sintering temperature). Higher sintering temperatures (from 1000 °C) stabilize the doped *M*:HAp phase with the general formula Ca_10_*M_x_*(PO_4_)_6_(OH)_2+2*x*_O_2*x*_. The incorporation of metallic cations is achieved by their insertion into the hexagonal channel of the hydroxyapatite structure, leading to low coordination of the doping element. EXAFS results confirm these observations, with short *M*–O distances. Specific characteristics were identified from the study. Iron (Fe^3+^) and cobalt (Co^3+^ translating the oxidation of the incorporated Co^2+^ species) cations move from the center of the hexagonal channel toward a shifted position, leading to a three-fold coordination which enables an increase in the inserted doping level. The cases of Ni^2+^ and Mn^2+^ (which evinces Mn^3+^ oxidation on heating) illustrate a more complicated mechanism leading to the general formula Ca_10−*x*−*y*_*M_x_*_+*z*_(PO_4_)_6_(OH)_2+2*y*−2*z*_O_2*y*−2*z*_ (with *y* < *z*) where *x*, *y*, and *z* represent calcium substitution, calcium site deficiency, and hexagonal channel insertion, respectively. Copper achieves a high doping level for sintering temperatures above 1100 °C, correlated with Cu^2+^ to Cu^+^ reduction. The insertion mechanism should be considered in order to properly manage sample syntheses and tune the biological behavior of the corresponding bioceramics. The sintering temperature can be used to prepare doped materials with quickly and easily available doping elements (when substituting calcium from the β-TCP phase) or long-term available doping elements (when inserted into the HAp phase).

## Figures and Tables

**Table 1:** Ionic radius (Å) of the different cations involved (LS: low spin, HS: high spin). Bold characters indicate cations and oxidation states incorporated in our syntheses.

Coordination	Mn^2+^		Mn^3+^		Fe^3+^		Co^2+^		Co^3+^	
	LS	HS	LS	HS	LS	HS	LS	HS	LS	HS
II	-	-	-	-	-	-	-	-	-	-
IV	-	0.66	-	-	-	0.49	-	0.58	-	-
VI	0.67	0.83	0.58	0.64	0.55	0.64	0.65	0.74	0.54	0.61
VII	-	0.90	-		-	-	-		-	
VIII	0.96		-		-	0.78	0.90		-	
IX	-		-		-		-		-	
Coordination	Ni^2+^		Cu^+^		Cu^2+^		Zn^2+^		Ca^2+^	
II	-		0.46		-		-		-	
IV	0.55 (0.49 *)		0.60		0.57 (0.57 *)		0.60		-	
VI	0.69		0.77		0.73		0.74		1.00	
VII	-		-		-		-		1.06	
VIII	-		-		-		0.90		1.12	
IX	-		-		-		-		1.18	

* Square coordination.
